# Role of Anti-Müllerian Hormone in pathophysiology, diagnosis and treatment of Polycystic Ovary Syndrome: a review

**DOI:** 10.1186/s12958-015-0134-9

**Published:** 2015-12-21

**Authors:** Agathe Dumont, Geoffroy Robin, Sophie Catteau-Jonard, Didier Dewailly

**Affiliations:** Service de Gynécologie Endocrinienne et de Médecine de la Reproduction, Hôpital Jeanne de Flandre, CHRU, 2 Avenue Eugène Avinée, 59037 Lille, France

**Keywords:** Polycystic Ovary Syndrome, Anti Müllerian Hormone, Hyperandrogenism, Ovulation induction

## Abstract

Polycystic ovary syndrome (PCOS) is the most common cause of chronic anovulation and hyperandrogenism in young women. Excessive ovarian production of Anti-Müllerian Hormone, secreted by growing follicles in excess, is now considered as an important feature of PCOS. The aim of this review is first to update the current knowledge about the role of AMH in the pathophysiology of PCOS. Then, this review will discuss the improvement that serum AMH assay brings in the diagnosis of PCOS. Last, this review will explain the utility of serum AMH assay in the management of infertility in women with PCOS and its utility as a marker of treatment efficiency on PCOS symptoms. It must be emphasized however that the lack of an international standard for the serum AMH assay, mainly because of technical issues, makes it difficult to define consensual thresholds, and thus impairs the widespread use of this new ovarian marker. Hopefully, this should soon improve.

## Background

Polycystic ovary syndrome (PCOS) is the most common cause of chronic anovulation and hyperandrogenism in young women and affects 5 to 10 % of the female population. Since 2003, the Rotterdam Consensus defines PCOS and considers the antral follicle count (AFC) on ultrasound as one of the diagnostic criteria. With the improvement in ultrasonographic technology, the number of follicles seen on ultrasound increases almost daily but remains dependent on the specific equipment. Serum AMH is synthetized by small antral follicles, which are precisely the ones seen on ultrasound. Serum AMH could therefore be used as a surrogate for the AFC in the diagnosis of PCOS. Serum AMH has also demonstrated its utility in the treatment of infertility. But the absence of an international standard for serum AMH assay and the inability to define thresholds makes application of serum AMH more difficult. The purpose of this review is to explain the relationship between AMH and PCOS and to describe the utility of serum AMH in the diagnosis and treatment of PCOS.

## AMH: from physiology to ovarian assessment

### Physiology of AMH

**Fig. 1:**
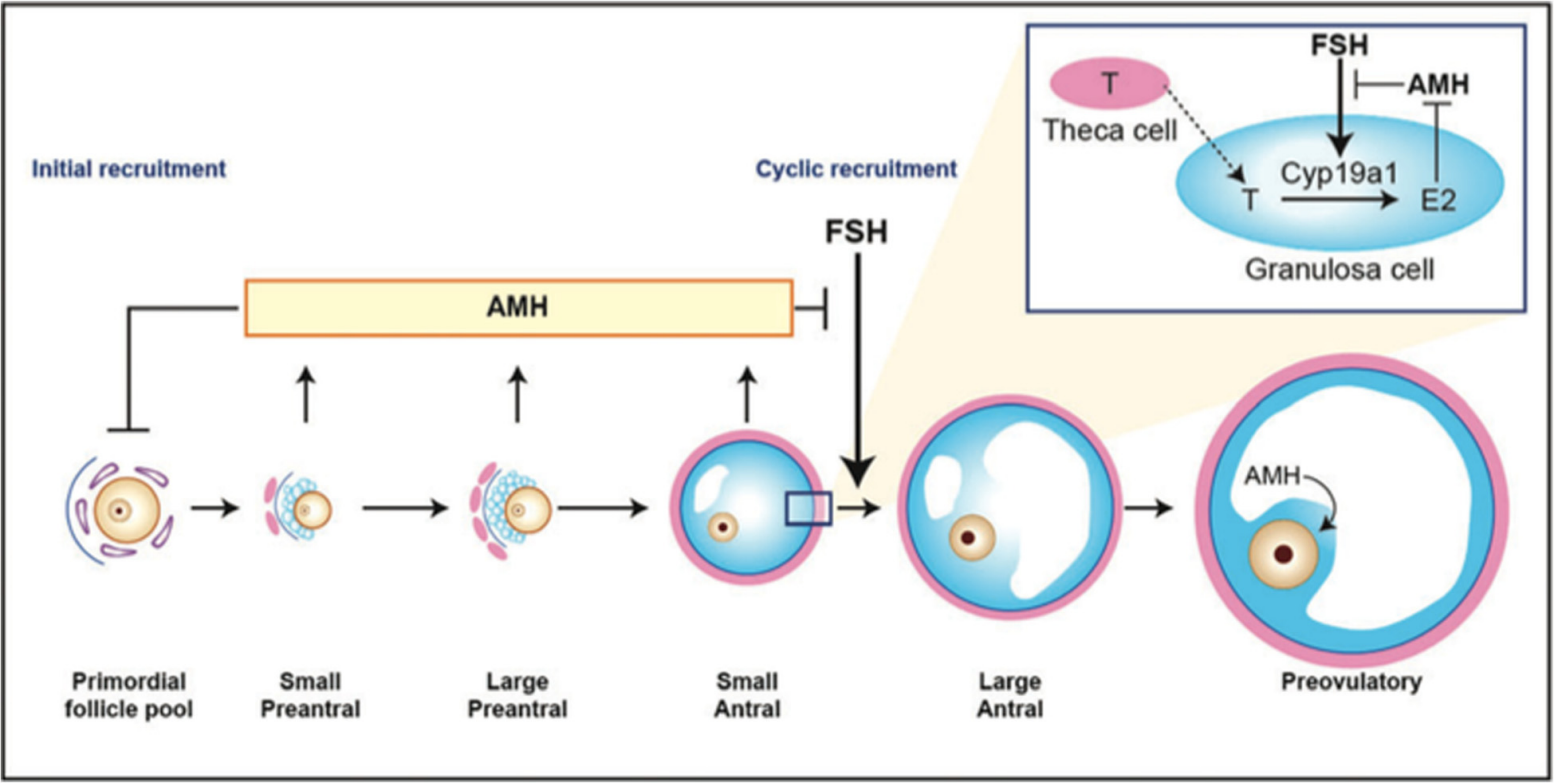
Schematic model of AMH actions in the ovary. Dewailly, D., et al., Hum Reprod Update, 2014 [24]. AMH, produced by the granulosa cells of small growing follicles, inhibits initial follicle recruitment and FSH-dependent growth and selection of pre antral and small antral follicles. In addition, AMH remains highly expressed in cumulus cells of mature follicles. The inset shows in more detail the inhibitory effect of AMH on FSH-induced CYP19a1 expression leading to reduced estradiol (E2) levels, and the inhibitory effect of E2 itself on AMH expression. T, testosterone; Cyp19a1, aromatase. Figure modified from van Houten et al. (2010)

AMH was only isolated and purified in 1984. Genes for AMH and its receptor were sequenced and cloned in 1986 and 1994 respectively [1]. AMH has been predominantly known for its role in male sexual differentiation [2]. In women, AMH expression is restricted to one cell type: the granulosa cells of the ovary. It starts around the 25th week of gestation continuing until menopause [1, 3]. AMH is expressed at all steps of folliculogenesis. It is initiated as soon as primordial follicles are recruited to grow into small preantral follicles and its highest expression is observed in pre antral and small antral follicles. AMH expression then decreases with the selection of follicles for dominance and is no longer expressed during the FSH dependent stages of follicular growth (except in the cumulus cells of pre ovulatory follicles), or in atretic follicles [4, 5] (Fig. 1).

The functional role of AMH in early follicular growth has been characterized by the study of “knocked out” models for the AMH gene (AMHKO) [5, 8–10]. When there is no AMH, primordial follicles are recruited faster, resulting in more growing follicles until the exhaustion of primary follicle pool at younger age than wild-type animals. AMH therefore has an inhibitory effect on early follicular recruitment preventing the entry of primordial follicles into the growing pool and thus premature exhaustion of follicles/oocytes [11]. AMH also has an inhibitory effect on cyclic follicular recruitment in vivo by reducing the follicle sensitivity to FSH. In vitro AMH inhibits FSH induced pre antral follicle growth [4, 5, 8, 9].

AMH also reduces the number of LH receptors in granulosa cells, also an FSH induced process [12]. Thus, it is clear that AMH is involved in the regulation of follicle growth initiation and in the threshold for follicle FSH sensitivity.

### AMH assay

In clinical application, AMH presents many opportunities but unfortunately there are difficulties due to several biological features of this molecule [13]. First, there is a molecular heterogeneity of the circulating AMH level with a non-cleaved biologically inactive form and a cleaved biologically active form [14, 15]. Also, there is variable sensitivity of the immunoassays to interference by complement C1q and C3 [16]. Then, the stability of AMH samples during the storage is not well known [17]. Moreover, AMH concentration varies according to the situation: in adults they are low (from 10.7 to 98 pmol/L) whereas in male new-borns they are very high (749 to 1930 pmol/L) [13]. Measurement thus involves different assays with different sensitivities. The last but not the least technical problem is the inter-laboratory variability, mainly for low values of serum AMH. The difficulty lies in the fact there are currently different ELISA immunoassays used worldwide: the Gen II (Beckman Coulter), the EIA AMH/MHS kits (IOT or “Immunotech” Beckman Coulter) and the AL-105-i (Anshlabs), which use different monoclonal antibody and different standards [18]. The lack of agreement between these assays explains the absence of consensual reference values and decision thresholds between teams in the literature [11]. To maximize the clinical utility of serum AMH measurement, current progress has been made: development of an ultrasensitive essay (‘pico AMH’ kit, Anshlabs), and automation on immuno-analyzers (Access Dxi automatic analyzer, Beckman Coulter. Cobas e instrument, Roche Diagnostics) allowing nearly identical values [19, 20]. Now that AMH assay has been automated, it is with great anticipation that an international standard for serum AMH will be developed.

### AMH as indicator of ovarian reserve and follicle growth

The ovarian reserve refers to the number of primordial follicles, defined at birth (around 1 million). This follicular capital decreases gradually throughout reproductive life, with the continuous initiation of growth of some follicles, and then mostly their apoptosis. There are about 400 000 follicles in adolescents’ ovaries (leading roughly to 400 ovulations), whereas only a thousand remains at the time of Menopause.

Serum AMH concentration is strongly correlated with the number of growing follicles since it represents AMH secretion from all developing follicles [21, 22]. Considering that the rate of initiation of follicle growth is deeply related to the initial follicular pool, we can assume that serum AMH is an indirect reflection of ovarian reserve. There is actually a very good correlation between serum AMH levels and ultrasonographic measure of the antral follicular count (AFC) [23, 24]. This can be explained because circulating AMH is mostly produced by granulosa cells of follicles from 2 to 9 mm in diameter (60 %), and those small follicles are precisely the ones counted on the ultrasound when the AFC is done [25]. Measurement of serum AMH is even more sensitive and specific than the AFC as it also reflects pre antral and small antral follicles (<2 mm), which are hardly seen in ultrasound. Serum AMH is therefore a deeper “probe” for the growing follicular pool than the AFC [6].

Serum AMH assay has many benefits over the other markers of ovarian reserve [11]. First, its plasmatic level is quite stable from one cycle to another and throughout the same cycle since the dominant follicle and corpus luteum do not secrete AMH [26, 27]. Conversely, the AFC and the FSH E2 pair have to be measured on the first 5 days of the cycle [28–30]. Also, a recent study has shown ethnicity does not influence serum AMH level, contrary to previous studies [31]. Besides its minor variability, serum AMH is also useful when the AFC cannot be done such as in obese, virgin or poorly echogenic patients [6]. Moreover, serum AMH level is rather independent from the hypothalamic pituitary axis and as such is not modified in pathologies such as hyperprolactinemia, functional hypothalamic amenorrhea or in incomplete and recent hypogonadotropic hypogonadism, providing serum FSH level remains normal or sub-normal [32]. The question thus actually arises about the replacement of the conventional markers of ovarian reserve by the AMH assay [33].

However, serum AMH can be influenced by many factors and some controversies persist. Obesity is often associated with a significantly lower level of serum AMH, but not in all studies [11, 34, 35]. There is also a controversy regarding the influence of hormonal contraception: to some authors, combined oestrogen progestin does not change AMH serum levels whereas others have recently reported a decrease of 29 to 50 % that could be explained by the suppression of gonadotropin secretion [36–40]. Similarly a decrease of serum AMH seven days after injection of depot leuprolide 3.75 mg, a GnRH agonist has been shown [41].

## Polycystic Ovary Syndrome (PCOS) and AMH

### Definition

Polycystic ovary syndrome (PCOS) is the most common cause of chronic anovulation and hyperandrogenism in young women and affects 5 to 10 % of the female population [42, 43]. PCOS is a diagnosis of exclusion and is defined by the Rotterdam classification (2003) [44] requiring at least 2 out of the 3 following characteristics: (i) cycle disorder, (ii) clinical or biological hyperandrogenism, (iii) antral follicular excess on ultrasound with ≥ 12 follicles from 2 to 9 mm per ovary and/or ovarian volume ≥ 10 ml. Metabolic disorders are often associated to PCOS (up to 50 %), including an increased rate of insulin resistance, regardless obesity [45]. PCOS thus imposes a considerable economic burden on national health systems [46]. PCOS is almost certainly a genetic condition but the precise causes of hyperandrogenism and anovulation, which are not always associated, are still under investigations [47, 48].

### Pathophysiology

PCOS is characterized by an increased number of follicles at all growing stages [49–51]. This increase is particularly seen in the pre-antral and small antral follicles, those which primarily produce AMH [52, 53]. Thus elevated serum AMH level, as a reflection of the stock of pre antral and small antral follicles, is 2–4 fold higher in women with PCOS than in healthy women [54–57] and is found in all PCOS populations [21, 22].

This increase in serum AMH was first thought to be only due to the higher number of pre antral and small antral follicles. However, production of AMH by granulosa cells was found in vitro to be 75-fold higher in anovulatory PCOS and 20-fold higher in normo ovulatory PCOS than in normal ovaries [55]. This suggests increased serum AMH levels in PCOS would also reflect an intrinsic dysregulation of the granulosa cells, in which AMH, itself, could be involved since an over expression of the AMH receptor type II (AMHRII) has also been demonstrated [58, 59].

The cause of such high production of AMH in antral follicles from PCO is currently unknown, but there is evidence to support a role played by androgens. Indeed, a positive correlation between serum androgen and AMH levels has been reported and the over production of androgens could be an intrinsic defect of thecal cells in PCOS [21, 22, 60–62].

**Fig. 2:**
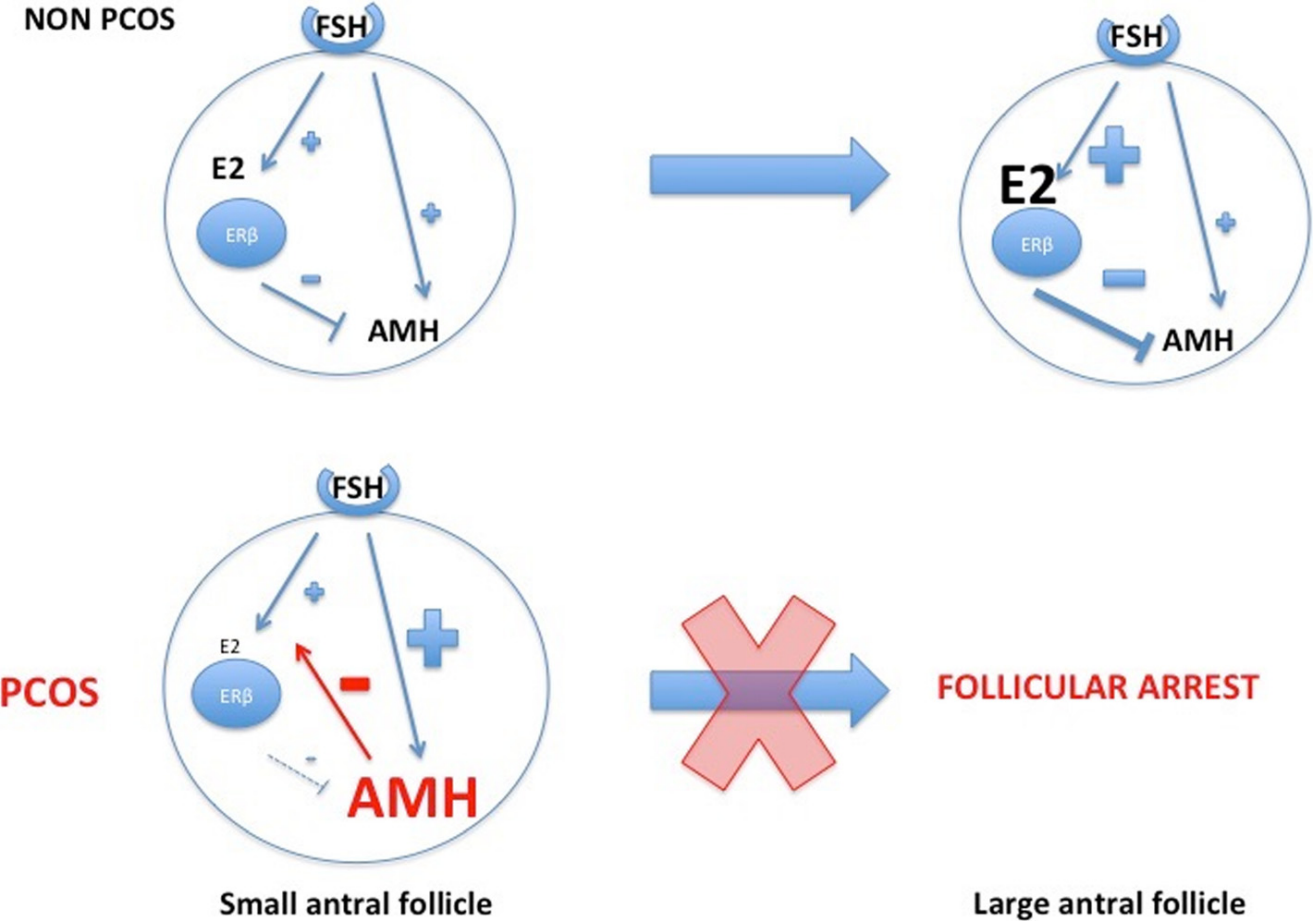
Schematic diagram of AMH regulation by FSH and E2 in GC of small and large antral follicles. Adapted from Grynberg M et al. JCEM, 2012 [65]. Until the small antral stage, AMH secretion is stimulated by different factors like FSH. Estradiol (E2) production under the influence of FSH is impaired by the inhibiting effect of AMH on aromatase. When estradiol concentration reaches a certain threshold in large antral follicles, it is capable of completely inhibiting AMH expression through ERβ, which predominates in growing follicles, thus overcoming the stimulation by FSH. In large follicles from PCO, the lack of FSH-induced E2 production and the high level of AMH impair the shift form the AMH to the E2 tone, thus leading to the follicular arrest

Studies demonstrated contradictory results concerning AMH regulation by gonadotropins. For some authors, gonadotropins (especially FSH) inhibit serum AMH production in vivo in normal ovaries [63, 64]. Pellat et al. [55] also demonstrated a reduced AMH production in granulosa cells from women with PCOS stimulated by FSH, but no such effect was found in ‘normal’ women. On the contrary, others demonstrated a stimulating effect of FSH on AMH expression in normal ovaries as well as in PCOS [65]. The recent finding that E2 inhibits AMH expression could reconcile those different results [66]: FSH may directly stimulate AMH in small antral follicles, as long as they do not express aromatase. But in larger follicles, by increasing E2 production with the recruitment of a dominant follicle, FSH would have an indirect inhibitory effect on AMH expression through the negative feedback of E2 (Fig. 2).

It has also been demonstrated that AMH significantly decreases not only the FSH receptor expression but also ovarian aromatase expression [18]. This allows protection of the small follicles from premature aromatase expression. However, when this protective effect exceeds its physiological role, because of AMH excess and/or because it lasts longer than it should in larger follicles, this could result in a defect in the selection of the dominant follicle, thus causing the so called the “follicular arrest”. The fact that AMH is inhibitory to FSH-dependent factors required for follicle dominance adds considerable significance to the high serum AMH expression found in PCOS and makes AMH a putative central actor of the ‘follicular arrest’. In good agreement, clinical studies have shown a relationship between high AMH and ovulatory disorder [55]. Likewise LH seems to stimulate AMH production by the granulosa cells in women with PCOS [55]. Acquisition of LH receptors by the granulosa cells happens sooner in PCOS [67]. In addition, some authors demonstrated that LH reduces AMHRII expression in granulosa luteal cells in normal ovaries and in women with normo-ovulatory PCOS, whereas it cannot do so in women with anovulatory PCOS [65, 68]. Besides LH stimulating effect on AMH expression, this lack of LH-induced down regulation of AMHRII expression in women with anovulatory PCOS could contribute to anovulation. Therefore, the premature action of LH might also contribute to the ‘follicular arrest’ through a mechanism involving the AMH system [67, 69].

To sum up, in PCOS, there are many abnormalities of folliculogenesis: (i) an increased number of small growing follicles [50], (ii) an inhibition of the terminal follicular growth [51], resulting in a lack of selection of the dominant follicle, so-called the “follicle arrest” [70], and (iii) a follicular apoptosis defect aggravating the excess of growing follicles [71, 72].

### Serum AMH in the diagnosis of PCOS

**Fig. 3:**
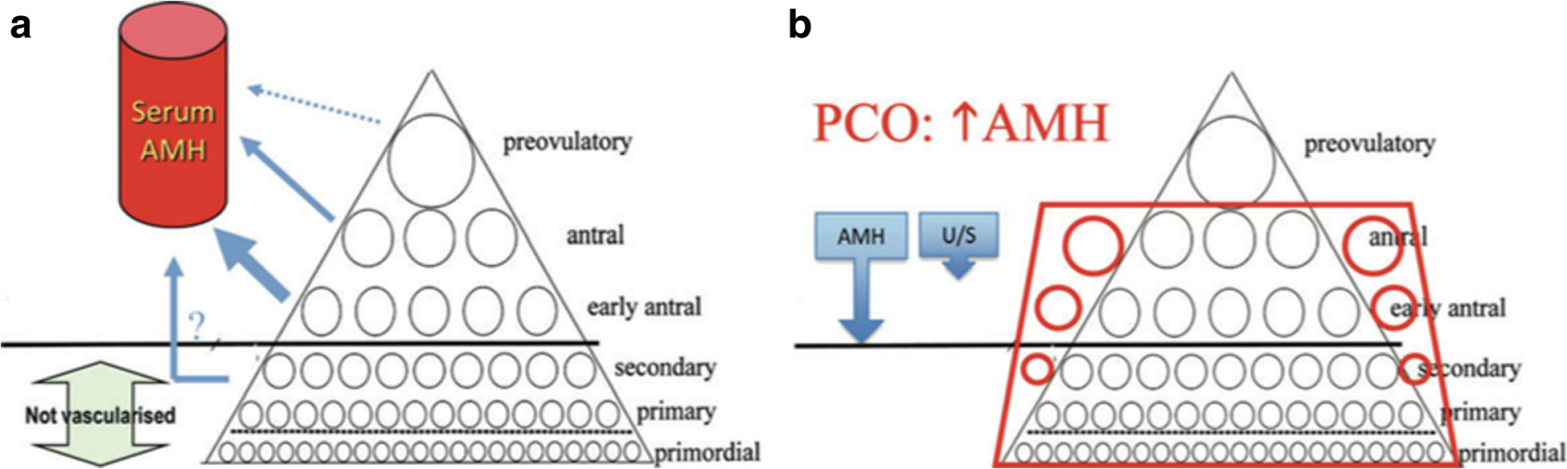
Rationale for the use of serum AMH assay as a probe for PCOM. Dewailly, D., et al., Hum Reprod Update, 2014 [24]. **a** All growing follicles secrete AMH but serum AMH reflects only the secretion from bigger follicles that are in contact with the vascular bed. As the numbers of follicles in all growth stages are strongly related to each other, serum AMH is considered to reflect the sum of growing follicles but not the number of primordial follicles that do not secrete AMH. **b** In PCO, the numbers of all growing follicles is increased, resulting in a marked increase in serum AMH level. AMH may be considered as a deeper and more sensitive probe to define follicle excess than the follicle count by ultrasound (U/S) since it appraises more follicle classes (blue arrows)

Given its strong implication in the pathophysiology of PCOS, serum AMH could be considered the “Gold Standard” in the diagnosis of PCOS. Even though serum AMH would be theoretically more accurate than AFC, as it reflects also the excess of small follicles non-visible on ultrasound [25, 6, 73] (Fig. 3), it is still considered premature to make this diagnostic transition.

The robust association between AMH and AFC has led some authors to compare their performance in the diagnosis of PCOS [74]. However, the results from the current literature are not homogeneous [11]. Part of this heterogeneity is due to the lack of well-defined population. In particular, some authors have used the PCOS definition established in 2003 at the Rotterdam conference, using 12 follicles of 2–9 mm diameter per ovary for the polycystic ovaries morphology (PCOM) [75]. This cut off is highly dependent on ultrasound equipment and operator skill, as demonstrated by Dewailly et al. [76]. Therefore, with the latest ultrasound generation, the threshold has evolved and is now up to 19 or 25 [73, 77, 78]. This threshold will probably continue to increase as newer ultrasound technologies and equipment are developed. Additionally, there are critical issues regarding what populations are included or excluded in the normative population. Lastly, there are technical issues regarding serum AMH assays leading to further heterogeneity of the results in the literature.

It is therefore impossible, so far, to propose a consensual and universal diagnostic threshold for serum AHM in the prediction of PCOS. However, in our experience, a cut off at 35 pmol/L (4.9 ng/mL) with the enzyme immunoassay AMH-EIA (EIA AMH/MIS kit) (“Immunotech”, ref A16507) provided by Beckman Coulter (France) had a good specificity (97 %) and a better sensitivity than the AFC (92 %) to distinguish women with PCOS from normal women [73]. This result was obtained after exclusion of women with asymptomatic PCO from the control group through cluster analysis, a mathematical procedure that avoids using predefined thresholds for AMH and AFC. This approach has been replicated in another setting [77]. Pigny et al. (unpublished data) have also compared the five serum AMH assays (as described above) for the diagnosis of PCOS. They proposed, with manual ELISAs assays, a higher cut-off at 5.6 ng/mL (40 pmol/L), as biological criteria indicative of PCOM, corresponding to the 95^th^ percentile of “pure” controls. They also proposed a threshold at 4.2 ng/mL (30 pmol/L) for the automatic assays (unpublished data). If confirmed with the new automatized serum AMH assays or the ultrasensitive assay, a high serum AMH level could then become a reliable and accurate marker for PCOM, and eventually replace the AFC, which also suffers from great controversy in the literature. As an ‘increased serum AMH’ does not refer to ovarian morphology, the acronym ‘PCOM’ would not be appropriate and we have therefore suggested the term “PCO-like abnormality” (i.e., PCOM and/or increased serum AMH) as the third item of the Rotterdam classification rather than ‘PCOM’ [79, 74].

Serum AMH level is also correlated to the severity of PCOS symptoms [68] and is higher when hyperandrogenism [62, 80] or oligo-anovulation is present [21, 55, 81]. By a principal component analysis, it has been shown a high serum AMH level can be considered as a marker of hyperandrogenism and could also be used as a substitute for this item in the Rotterdam classification [82].

This would reconcile the different classifications currently available for PCOS because some require hyperandrogenism as a necessary criterion [56]. We thus propose the following strategy: for the diagnosis of PCOS, hyperandrogenism and oligo anovulation should be first sought, after excluding all alternative diagnoses. If one is missing, a high AFC or/and AMH level could be used instead.

In adolescent and young women with PCOS, it is sometimes difficult to evaluate the ovaries on ultrasonography. It can also be difficult to estimate the share of the physiological and pathological, concerning acne and cycle disorders. Serum AMH assay is therefore a true alternative, as it is recommended by the American association of clinical endocrinologists [83]. Adolescents with PCOS have higher serum AMH levels, with a thresholds set at 30 pmol/L [84], which is in the range of values found in the literature for older PCOS women [85, 86].

However, it must be noticed that the thresholds for excessive AFC or high serum AMH level have to be reviewed and validated worldwide, since recent technical developments in ultrasound and assays could lead to their modification. In the meantime, it is recommended that each centre set its own thresholds.

## THE use of serum AMH for the treatment of PCOS

### AMH: useful in treatments of infertility?

Because of the dysovulation often associated, PCOS is a frequent cause of infertility. The follicular excess present in this pathology can be responsible for an ovarian over-response when induction ovulation treatments are used increasing risk for ovarian hyperstimulation syndrome (OHSS). This is a challenging situation as the minimal effective dose is often very close to the overdose leading to hyperstimultation.

Thus, in addition to its diagnostic role, serum AMH level is useful to establish treatment protocols and to define the best strategy for ovulation induction in infertile women with PCOS.

#### Clomiphene citrate

So far, there are very few studies that have examined the predictive power of serum AMH level in the response to clomiphene citrate (CC). Mahran et al. [87] have proposed a threshold at 3.4 ng/mL above which a resistance to CC is highly expected, suggesting a higher starting dose should be used. However, the free androgen index was significantly higher among the non-responding patients which may have biased the results since this parameter is a well-known negative predictor. Also the sensitivity and specificity were poor (around 75 %), leading to wrong information in ¼ of cases, making difficult the use of this threshold in clinical practice.

#### Recombinant FSH

Serum AMH level appears to be a good predictive marker for the risk of OHSS [88], mostly occurring in PCOS, as proved in the meta-analysis of Broer et al. [89]. However the establishment of an accurate threshold remains difficult because of the heterogeneity of the OHSS definition and technical issues with the AMH assays.

To conclude, serum AMH determination may be a helpful tool in the prediction of the ovarian response to gonadotropins in PCOS. Difficulties persist, however, since there is no consensus on the threshold for the AMH values. This is due to the well-known difficulty of quantification and standardization of the AMH assays.

#### Ovarian drilling

Laparoscopic ovarian drilling (LOD) is currently recommended as a successful second line treatment for ovulation induction in women with PCOS. It is considered to be an alternative to gonadotropin stimulation in the case of CC resistance [90].

The aim is to trigger spontaneous ovulation by destroying small amounts of ovarian cortex. The physiological mechanism remains unknown, but drilling significantly alters the hormonal environment within the ovary. The utility of the AMH assay as a predictor for LOD outcome has been recently questioned [91]. Two studies evaluated serum AMH before LOD to correlate with the results in terms of spontaneous ovulation [92, 93]. Elsmashad et al. [92] evaluated the effect of LOD, in PCOS, on serum AMH and ovarian stromal blood flow changes, by using three-dimensional power Doppler ultrasonography. They showed LOD was followed by lower serum AMH levels and less elevated Doppler flow indices. Amer et al. [93] also showed that women who ovulated after LOD had lower pre-operative AMH levels. They identified a pretreatment AMH level cut-off of 7.7 ng/mL (sensitivity 78 %, specificity 76 %), which predicted failure of LOD. Weaknesses of this study included the small sample size and the fact LOD was used as a first line method for ovulation induction

### AMH: a marker of treatment efficiency on PCOS symptoms?

To normalize the menstrual cycle, it is common to prescribe a hormonal contraception to women with PCOS. As explained above, there is, however, controversy regarding the impact of hormonal contraception on the AMH circulating level [6, 38]. In PCOS, it has recently been shown [94] that in current users of hormonal contraception, SHBG was increased, leading to a diminished bioavailable androgen level, and that AMH and AFC levels were decreased. However, no study has reported so far this decrease of serum AMH has a predictive value on the subsequent fertility.

To have an anti-androgenic effect and thus decrease the clinical hyperandrogenism (such as acne, hirsutism) it is common, in PCOS, to use oral combined contraceptive treatment sometimes combined with antiandrogens such as drospirenone or cyproterone acetate (CPA) or spironolactone [95, 96]. Panidis et al. [97] have shown a significant decrease in serum AMH levels in women with PCOS with ethinylestradiol + CPA but not with use of ethinylestradiol + drospirenone.

Some studies have evaluated the relationship between weight loss, menstrual cyclicity and serum AMH levels in overweight women with PCOS. Moran et al. [98] showed better menstrual improvement after weight loss in women with lower baseline serum AMH. Serum AMH could therefore be used as a potential predictive factor of cycle normalization after weight loss. Nybacka et al. [99] described a significant decrease in serum AMH levels after diet in obese women with PCOS. Thomson et al. [100] demonstrated long-term weight loss (twenty weeks) resulted in improvements in reproductive function but did not change serum AMH levels. Lastly, various effects on AMH level were obtained with Metformin usage in women with PCOS [97, 101–104].

Altogether, no threshold for serum AMH can be proposed to predict the effectiveness of these treatments.

## Conclusion

It is now undeniable that serum AMH is a valuable tool for the diagnosis of PCOS. As for its benefit in the treatment of PCOS, there may be an advantage in therapeutic decision support, but this needs to be confirmed by further studies. However, the current technical difficulties to set up consensual serum AMH thresholds [105, 19] (stability and heterogeneity of circulating AMH, wide range of values, inter laboratory variability, different immunoassays used worldwide) may have curbed the enthusiasm of some clinicians to make it “THE” marker of PCOM. However, we must remain optimistic regarding the latest progress made, making it more and more realistic that this assay may, one day, replace the AFC in the Rotterdam classification.
